# Synthesis and characterization of some novel polythiophene derivatives containing pyrazoline

**DOI:** 10.1080/15685551.2022.2086413

**Published:** 2022-06-09

**Authors:** Vu Quoc Trung, Tran Thi Thuy Duong, Nguyen Thi Dua, Nguyen Ngoc Linh, Lai Dang Cuong, Dao Phuong Thao, Vo Khac Huy, Nguyen Hoang Ha Phuong, Nguyen Hien, Duong Khanh Linh, Vu Quoc Manh, Nguyen Thuy Chinh, Thai Hoang, Luc Van Meervelt

**Affiliations:** a Faculty of Chemistry, Hanoi National University of Education, Hanoi, Vietnam; b Tay Ho High School, Hanoi, Vietnam; c Faculty of Pharmacy, Thanh Do University, Hanoi, Vietnam; d Bien Hoa Gifted High School, Phu Ly, Vietnam; e HUS High School for Gifted Student, Hanoi, Vietnam; f Vinschool The Harmony High School, Hanoi, Vietnam; g Institute for Tropical Technology, Vietnam Academy of Science and Technology, Hanoi, Vietnam; h Vietnam Academy of Science and Technology, Graduate University of Science and Technology, Hanoi, Vietnam; i Department of Chemistry, KU Leuven, Biomolecular Architecture, Leuven (Heverlee), Belgium

**Keywords:** Polythiophene derivatives, pyrazoline heterocycle, chemical polymerization, crystal structure

## Abstract

Eight polythiophene derivatives containing pyrazoline side groups were synthesized by a chemical oxidative coupling polymerization using FeCl_3_. The crystal structures of four monomers were determined which confirm the almost perpendicular orientation of the thiophene and pyrazoline rings, while the other substituents are more coplanar. Analyses of IR, ^1^H-NMR, Raman and UV-Vis spectra demonstrated that the suggested polymerization was successful to generate the synthesized polythiophenes with the expected structures. The morphology of the synthesized polythiophenes was studied by SEM. The different substituents attached to the 1- and 3-positions of the pyrazoline side chain led to differences in optical properties, electrical conductivity, and thermal stability of the synthesized polythiophenes. By adding a pyrazoline side chain to polythiophenes, some polymers achieve good solubility, electrical conductivity of about 1.3 × 10^–6^ S/cm, high fluorescence intensity (above 40,000 a.u.) at 505–550 nm and thermal stability up to 590°C in the air.

## 1. Introduction

Recently, polymers containing heteroaromatic rings have been commonly synthesized and characterized because of their effective applicability in many optoelectronic devices [1-5]. Among these polymers, polythiophene and its derivatives are an important representative class of π-conjugated polymers based on their high environmentally and thermally stability, and easy control of conductivity, electrochemical and optical properties by side chain modification [2,6]. By increasing alkoxy or alkyl substituent length at the 3-position of the thiophene ring, properties of polythiophenes have been significantly improved for applications in electronics. The incorporation of heteroaromatic rings into polythiophenes improves extensively their characteristics and their solubility in many organic solvents [7–10].

Most polythiophenes have exhibited outstanding optical characteristics such as biochromism [11], thermochromism [12,13], photochromism [14] and ionochromism [15]. The optoelectronic characteristics of polythiophene derivatives make them remarkable candidates for polymer light emitting diodes [16,17], organic field-effect transistors [15,18], organic photovoltaic cells [19,20], biomedical applications [21], and other related applications [22,23]. A broader application area of polythiophenes is water-soluble sensors for the detection of small bioanalytes, DNAs, proteins and metal ions [24–29].

Many nitrogen-containing heterocycles such as quinoxalines, 1,3,5-triazines, 1,2,4-triazoles, … have been added on polythiophenes as side chains to modify the polymer main chain conformation and the electron density of the π-conjugated polymers [6]. For example, a series of polythiophenes containing benzothiazole were synthesized by Radhakrishnan *et al*. using chemical oxidation polymerization [30–32]. In addition, Seok H. A. *et al*. synthesized some soluble polythiophenes containing an electron transporting benzotriazole moiety and characterized their electroluminescence efficiency in light-emitting diodes [33]. Moreover, in our earlier studies, Vu *et al*. synthesized and characterized novel polythiophene derivatives bearing 1,3-thiazolidin-4-one or 1,2,4-triazole heterocycles with good solubility in some organic solvents [34–36].

This study presents the synthesis and characterization of a new series of polythiophene derivatives containing pyrazoline at the C-3-position of the thiophene ring. The crystal structures of all monomers were determined using X-ray diffraction analyses. Based on FeCl_3_ -mediated chemical oxidation polymerization of the monomer, the polythiophene derivatives were synthesized and structurally studied by SEM, TGA methods, UV-Vis spectra, photoluminescence spectra and electrical conductivity.

## 2. Materials and methods

### 2.1. Materials

Analytical grade chemicals: DMSO, 3-thiophenecarbaldehyde, acetophenone, 4-bromoacetophenone, 4-methylacetophenone, 4-methoxyacetophenone, 4-chloroacetophenone, 4-ethoxyactophenone, phenylhydrazine, thiosemicarbazide, iron(III) chloride, potassium hydroxide, hydrochloric acid, ethanol, chloroform from Sigma Aldrich were used without any further purification. NMR samples were prepared in conventional deuterated solvents such as DMSO-*d_6_*, CDCl_3_, and CD_3_OD.

### 2.2. Devices and methods

A Gallenkamp melting point apparatus was used to measure the melting points of the monomers. A Nicolet Impact 410 FT-IR Spectrometer was used with a pellet of sample mixed with KBr to measure the IR spectra. ^1^H-NMR spectra were recorded using a Bruker XL-500 Spectrometer operating at 500 MHz using CDCl_3_ and DMSO-*d_6_* as solvents with the data given in parts per million (ppm) and spin–spin coupling constants (*J*) given in Hz. UV-Vis spectra of solid polymers were determined using a Jasco V670 UV-Vis Spectrophotometer. A Shimadzu Simultaneous Measuring Instrument, DTG–60/60 H was used with a heating speed of 10°C/min from 30°C to 600°C in the air to record the TGA/DTA thermograms. A SEM–Hitachi–4800 was used to perform the SEM analysis of the polymers. The Agilent E4980A Precision LCR Meter (United States) was used to determine the conductivity with polymer tablets of 0.5 cm diameter.

### 2.3. Synthesis

#### 2.3.1. Synthesis of eight monomers containing pyrazoline (1a–1d, 2a–2d)

##### 2.3.1.1. Synthesis of four α,β-unsaturated ketone compounds *(a–d)*

To a mixture of thiophene-3-carbaldehyde (0.1 mol) and an acetophenone derivative (0.1 mol) dissolved in 100 mL of ethanol was added 10 mL of 50% KOH solution. At room temperature, the mixture was stirred for 5 hours until the precipitate appeared. After filtration, the products **a–d** were achieved as solids, which were recrystallized from hot ethanol.

The morphology, molecular formula, molecular mass and melting points were determined. The crystal and molecular structures of compounds **c** and **d** were summarized in our previous study [7].

*3-(3-phenylprop-1-ene-3-one-1-yl)thiophene* (**a**): Yellow solid; yield 85%; m.p. 80 °C; FT-IR (KBr, cm^–1^): 2974 (CH aromatic, alkene), 1659 (C = O), 1597 (C = C, C = N), 1017 (–CH = trans). ^1^H-NMR [500 MHz, *d*_6_-CDCl_3_, ppm, *J* (Hz)]: 7.38 (dd, *J* = 3.0, *J* = 5.0, **H**2), 7.42 (d, *J* = 4.0, **H**4), 7.57 (dd, *J* = 3.0, **H**5), 7.79 (d, *J* = 15.5, **H**6), 7.34 (d, *J* = 16.0, **H**7), 7.99 (d, *J* = 8.5, **H**10 and **H**10’), 7.49 (d, *J* = 8.5, **H**11 and **H**11’), 7.6 (m, *J* = 3.0, **H**12). ^13^C-NMR [125 MHz, *d_6_*-CDCl_3_, ppm]: 125.26 (**C**2), 128.62 (**C**3), 128.45 (**C**4), 127.06 (**C**5), 132.70 (**C**6), 129.09 (**C**7), 190.83 (**C**8), 138.31 (**C**9), 138.28 (**C**10, **C**10’), 138.22 (**C**11, **C**11’), 121.93 (**C**12). Calculation for C_13_H_10_OS: M = 214 au.

*3-(3-(4-methylphenyl)prop-1-ene-3-one-1-yl)thiophene* (**b**): Yellow solid; yield 85%; m.p. 80 °C; FT-IR (KBr, cm^–1^): 3082 (CH alkane), 2974 (CH aromatic, alkene), 1652 (C = O), 1595 (C = C, C = N), 1030 (–CH = trans). ^1^H-NMR [500 MHz, *d*_6_-CDCl_3_, ppm, *J* (Hz)]: 2.43 (s, C**H**_3_), 7.29 (d, *J* = 8.0, **H**11 and **H**11’), 7.34 (d, *J* = 15.5, **H**7), 7.37 (dd, *J* = 3.0, *J* = 5.0, **H**2), 7.42 (d, *J* = 4.0, **H**4), 7.59 (dd, *J* = 3.0, **H**5), 7.78 (d, *J* = 15.5, **H**6), 7.91 (d, *J* = 8.5, **H**10 and **H**10’). ^13^C-NMR [125 MHz, *d_6_*-CDCl_3_, (ppm)]: 125.29 (**C**2), 128.86 (**C**3), 128.60 (**C**4), 126.98 (**C**5), 135.72 (**C**6), 129.32 (**C**7), 190.29 (**C**8), 143.54 (**C**9), 138.32 (**C**10, **C**10’), 137.85 (**C**11, **C**11’), 121.94 (**C**12), 21.67 (**C**H_3_). Calculation for C_14_H_12_OS: M = 228 au.

##### 2.3.1.2. Synthesis of eight monomers containing pyrazoline (1a–1d, 2a–2d)

To a mixture of phenylhydrazine (3 mmol) and an *α,β-unsaturated ketone compound* **a–d** (1 mmol) dissolved in 20 mL of ethanol were added 5 drops of concentrated HCl solution. The reaction mixture was refluxed for 8 hours at 100°C. The precipitated solids were filtered off and recrystallized from hot ethanol to give the four monomers containing pyrazoline **1a–1d**.

To a mixture of thiosemicarbazide (3 mmol) and an *α,β-unsaturated ketone compound* **a–d** (1 mmol) dissolved in 15 mL of ethanol were added 5 drops of concentrated HCl. The reaction mixture was refluxed for 8 hours at 100°C. The precipitate was filtered off and recrystallized from ethanol to give the four monomers containing pyrazoline **2a–2d**.

*1,3-bisphenyl-5-(thiophen-3-yl)-2-pyrazoline* (**1a**): White solid, yield 67%; m.p. 190°C, FT-IR (KBr, cm^–1^): 3096 (CH aromatic, alkene), 2928 (CH alkane), 1593 (C = C), 1499 (C = N). ^1^H-NMR [500 MHz, *d*_6_-CDCl_3_, ppm, *J* (Hz)]: 3.19 (m, 1H, *J* = 5.0, *J* = 17.0, **H**4b), 3.75 (m, *J* = 7.0, *J* = 16.0, **H**4a), 5.4 (dd, *J* = 5.0, J = 7.0, **H**5), 6.80 (t, 1H, *J* = 7.5, **H**7), 7.17 (m, *J* = 2.0, **H**8), 7.01 (dd, *J* = 1.5, *J* = 5.0, **H**10), 7.73 (m, *J* = 9.0, **H**12 and **H**12’), 6.39 (m, *J* = 9.0, **H**13 and **H**13’), 7.33 (dd, **H**14), 7.59 (d, *J* = 8.5, **H**16 and **H**16’), 7.10 (d, *J* = 8 Hz, **H**17 and **H**17’), 7.29 (dd, *J* = 3.0, J = 8.0, **H**18). ^13^C-NMR [125 MHz, *d_6_*-CDCl_3_, ppm]: 146.70 (**C**3), 42.78 (**C**4), 51.24 (**C**5), 125.31 (**C**6), 125.09 (**C**7), 123.01 (**C**8), 120.74 (**C**10), 128.18 (**C**11), 140.01 (**C**12, **C**12’), 128.18 (**C**13, **C**13’), 142.67 (**C**14), 140.04 (**C**15), 128.05 (**C**16, **C**16’), 127.36 (**C**17, **C**17’), 125.09 (**C**18). Calculation for C_19_H_16_N_2_S: M = 304 au.

*3-(4-methylphenyl)-1-phenyl-5-thiophenyl-2-pyrazoline* (**1b**): White crystal, yield 70%; m.p. 200°C. ^1^H-NMR [500 MHz, *d*_6_-CDCl_3_, ppm, *J* (Hz)]: 3.15 (m, *J* = 5.0, *J* = 17.0, **H**4b), 3.75 (m, *J* = 7.0, *J* = 16.0, **H**4a), 5.35 (dd, *J* = 5.0, *J* = 7.0, **H**5), 6.79 (m, *J* = 7.5, **H**7), 7.16 (m, *J* = 2.0, **H**8), 7.02 (dd, *J* = 1.5, *J* = 7.5, **H**10), 7.51 (m, *J* = 9.0, **H**12 and **H**12’), 7.0 (m, *J* = 9.0, **H**13 and **H**13’), 7.59 (d, *J* = 8.5, **H**16 and **H**16’), 7.10 (d, *J* = 8.0, **H**17 and **H**17’), 7.29 (dd, *J* = 3.0, J = 8.0, **H**18), 3.4 (s, C**H**_3_). ^13^C-NMR [125 MHz, *d_6_*-CDCl_3_, ppm]: 147.40 (**C**3), 42.73 (**C**4), 60.51 (**C**5), 125.75 (**C**6), 121.04 (**C**7), 119.14 (**C**8), 113.54 (**C**10), 129.99 (**C**11), 138.75 (**C**12, **C**12’), 129.26 (**C**13, **C**13’), 145.25 (**C**14), 143.52 (**C**15), 128.88 (**C**16, **C**16’), 127.0 (**C**17, **C**17’), 125.59 (**C**18), 21.38 (**C**H_3_). Calculation for C_20_H_18_N_2_S: M = 318 au.

*3-(4-methoxyphenyl)-1-phenyl-5-thiophenyl-2-pyrazoline* (**1c**): White crystal, yield 75%; m.p. 215°C; FT-IR (KBr, cm^–1^): 3043 (CH aromatic, alkene), 2962, 2836 (CH alkane), 1594 (C = C), 1496 (C = N). ^1^H-NMR [500 MHz, *d*_6_-CDCl_3_, ppm, *J* (Hz)]: 3.1 (m, *J* = 5.0, *J* = 17.0, **H**4b), 3.7 (m, *J* = 7.0, *J* = 16.0, **H**4a), 5.3 (dd, *J* = 5.0, *J* = 7.0, **H**5), 6.78 (t, *J* = 7.5, **H**7), 7.01 (dd, *J* = 1.5, *J* = 5.0, **H**8), 7.16 (m, *J* = 2.0, **H**10), 7.66 (m, *J* = 9.0, **H**12 and **H**12’), 6.91 (m, *J* = 9.0, **H**13 and **H**13’), 7.19 (d, *J* = 8.5, **H**16 and **H**16’), 7.09 (d, *J* = 8.0, **H**17 and H17’), 7.28 (dd, *J* = 3.0, *J* = 8.0, **H**18), 3.83 (s, OC**H**_3_). ^13^C-NMR [125 MHz, *d_6_*-CDCl_3_, ppm]: 147.25 (**C**3), 55.35 (**C**4), 60.55 (**C**5), 125.54 (**C**6), 121.03 (**C**7), 119.02 (**C**8), 113.47 (**C**10), 143.56 (**C**11), 145.4 (**C**12, **C**12’), 128.87 (**C**13, **C**13’), 145.40 (**C**14), 143.56 (**C**15), 128.87 (**C**16, **C**16’), 127.25 (**C**17, **C**17’), 125.61 (**C**18), 42.84 (O**C**H_3_). Calculation for C_20_H_18_N_2_OS: M^+^ = 334,9 au.

*3-(4-bromophenyl)-1-phenyl-5-thiophenyl-2-pyrazoline* (**1d**): White crystal, yield 80%; m.p. 220°C. FT-IR (KBr, cm^–1^): 3052 (CH alkane), 2928 (CH aromatic, alkene),1596 (C = C, C = N). ^1^H-NMR [500 MHz, *d*_6_-CDCl_3_, ppm, *J* (Hz)]: 3.1 (m, *J* = 5.0, *J* = 17.0, **H**4b), 3.7 (m, *J* = 7.0, *J* = 16.0, **H**4a), 5.4 (dd, *J* = 5 Hz, *J* = 7 Hz, **H**5), 6.8 (t, *J* = 7.5, **H**7), 7.16 (m, *J* = 2.0, **H**8), 7.0 (dd, *J* = 1.5, *J* = 7.5, **H**10), 7.51 (m, *J* = 9.0, **H**12 and **H**12’), 7.59 (m, *J* = 9.0, **H**13 and **H**13’), 7.59 (d, *J* = 8.5 Hz, **H**16 and **H**16’), 7.10 (d, *J* = 8.0, **H**17 and **H**17’), 7.29 (dd, *J* = 3.0, *J* = 8.0, **H**18). ^13^C-NMR [125 MHz, *d_6_*-CDCl_3_, ppm]: 145.98 (**C**3), 42.43 (**C**4), 60.64 (**C**5), 121.16 (**C**6), 122.56 (**C**7), 119.56 (**C**8), 113.62 (**C**10), 128.64 (**C**11), 131.71 (**C**12, **C**12’), 131.73 (**C**13, **C**13’), 144.75 (**C**14), 143.12 (**C**15), 127.19 (**C**16, **C**16’), 127.15 (**C**17, **C**17’), 125.46 (**C**18). Calculation for C_19_H_15_BrN_2_S: M = 383 au.

*1-carbothiamide-3-phenyl-5-thiophenyl-2-pyrazoline* (**2a**): White solid, yield 65%; m.p. 184°C. FT-IR (KBr, cm^–1^): 3472 (NH_2_), 3091.7 (CH alkane), 2953 (CH aromatic, alkene), 1575 (C = C, C = N). ^1^H-NMR [500 MHz, *d*_6_-CDCl_3_, ppm, *J* (Hz)]: 3.3 (dd, *J* = 3.0, *J* = 17.5, **H**4b), 3.7 (dd, *J* = 11.0, *J* = 17.5, **H**4a), 6.2 (dd, *J* = 3.0, *J* = 11.0, **H**5), 6.9 (d, *J* = 5.0, **H**7), 7.26 (m,1H, *J* = 3.0, **H**8), 7.2 (d, *J* = 2.0, **H**10), 7.46 (dd, *J* = 7.0, **H**12 and **H**12’), 7.74 (dd, *J* = 7.0, **H**13 and **H**13’), 7.43 (m, *J* = 6.5, **H**14). ^13^C-NMR [125 MHz, *d_6_*-CDCl_3_, ppm]: 156.35 (**C**3), 42.12 (**C**4), 59.40 (**C**5), 141.91 (**C**6), 125.32 (**C**7), 126.51 (**C**8), 121.81 (**C**10), 130.67 (**C**11), 131.09 (**C**12, **C**12’), 126.96 (**C**13, **C**13’), 128.90 (**C**14), 176.71 (**C**15). Calculation for C_14_H_13_N_3_S_2_: M = 287 au.

*1-carbothiamide-3-(4-methylphenyl)-5-thiophenyl-2-pyrazoline* (**2b**): White solid, yield 65%; m.p. 184°C. FT-IR (KBr, cm^–1^): 3472 (NH_2_), 3095 (CH alkane), 2916 (CH aromatic, alkene), 1574 (C = C, C = N). ^1^H-NMR [500 MHz, *d*_6_-CDCl_3_, ppm, *J* (Hz)]: 3.25 (dd, *J* = 3.0, *J* = 17.5, **H**4b), 3.73 (dd, *J* = 11.0, *J* = 17.5, **H**4a), 6.17 (dd, *J* = 3.0, *J* = 11.0, **H**5), 6.98 (dd, *J* = 1.5, *J* = 5.0, **H**7), 7.23 (m, *J* = 3.0, **H**8), 7.2 (d, *J* = 2.5, **H**10), 7.25 (dd, *J* = 7.5, **H**12 and **H**12’), 7.62 (d, *J* = 8.0, **H**13 and **H**13’), 1.65 (s, C**H**_3_). ^13^C-NMR [125 MHz, *d_6_*-CDCl_3_, ppm]: 156.47 (**C**3), 42.12 (**C**4), 59.29 (**C**5), 141.96 (**C**6), 125.34 (**C**7), 126.42 (**C**8), 121.75 (**C**10), 129.58 (**C**11), 127.85 (**C**12, **C**12’), 126.93 (**C**13, **C**13’), 141.63 (**C**14), 176.43 (**C**15), 21.54 (**C**16). Calculation for C_15_H_15_N_3_S_2_: M = 301 au.

*1-carbothiamide-3-(4-methoxyphenyl)-5-thiophenyl-2-pyrazoline* (**2c**): Orange crystal, yield 87%; m.p. 190°C. FT-IR (KBr, cm^–1^): 3433 (NH_2_), 3266 (CH alkane), 2833 (CH aromatic, alkene), 1610 (C = C, C = N); ^1^H-NMR [500 MHz, *d*_6_-CDCl_3_, ppm, *J* (Hz)]: 3.2 (dd, *J* = 3.0, *J* = 17.5, **H**4b), 3.7 (dd, *J* = 11.5, *J* = 17.5, **H**4a), 6.1 (dd, *J* = 3.0, *J* = 11.0, **H**5), 6.98 (dd, *J* = 1.5, *J* = 5.0, **H**7), 7.25 (m, *J* = 2.0, *J* = 5.0, **H**8), 7.2 (dd, *J* = 1.5, *J* = 2.0, **H**10), 7.67 (m, *J* = 9.0, **H**12 and **H**12’), 6.94 (m, *J* = 9.0, **H**13 and **H**13’), 3.85 (s, OC**H**_3_). ^13^C-NMR [125 MHz, *d_6_*-CDCl_3_, ppm]: 156.25 (**C**3), 42.17 (**C**4), 59.53 (**C**5), 142.03 (**C**6), 125.38 (**C**7), 126.42 (**C**8), 121.75 (**C**10), 123.18 (**C**11), 128.69 (**C**12, **C**12’), 114.35 (**C**13, **C**13’), 162.00 (**C**14), 176.19 (**C**15), 55.48 (**C**16). Calculation for C_15_H_15_N_3_OS_2_: M^+^ = 317 au.

*1-carbothiamide-3-(4-bromophenyl)-5-thiophenyl-2-pyrazoline* (**2d**): White crystal, yield 90%; m.p. 210°C. FT-IR (KBr, cm^–1^): 3470 (NH_2_), 3039 (CH alkane), 2922 (CH aromatic, alkene),1574 (C = C, C = N). ^1^H-NMR [500 MHz, *d*_6_-CDCl_3_, ppm, *J* (Hz)]: 3.2 (dd, *J* = 3.5, *J* = 17.5, **H**4b), 3.7 (dd, *J* = 11.5, *J* = 17.5, **H**4a), 6.18 (dd, *J* = 3.0, *J* = 11.0, **H**5), 6.97 (d, *J* = 5.0, **H**7), 7.27 (dd, *J* = 2.0, *J* = 5.0, **H**8), 7.2 (d, *J* = 2.0, **H**10), 7.57 (dd, *J* = 8.5, **H**12 and **H**12’), 7.60 (dd, *J* = 8.5, **H**13 and **H**13’). ^13^C-NMR [125 MHz, *d_6_*-CDCl_3_, ppm]: 155.22 (**C**3), 41.98 (**C**4), 59.53 (**C**5), 141.76 (**C**6), 125.22 (**C**7), 126.63 (**C**8), 121.84 (**C**10), 132.18 (**C**11), 139.60 (**C**12, **C**12’), 125.55 (**C**13, **C**13’), 128.33 (**C**14), 176.79 (**C**15). Calculation for C_14_H_12_BrN_3_S_2_: M^+^ = 365,7 au.

#### 2.3.2. Synthesis of eight polythiophene derivatives containing pyrazoline (3a–3d, 4a–4d)

The polymerization mixture of each monomer **1a–1d, 2a–2d** (0.1 mol) and anhydrous iron(III) chloride (0.4 mol) in 50 mL of dry chloroform was stirred for 24 hours at room temperature under nitrogen atmosphere [6,24]. After filtration, the precipitate was purified by washing several times with deionized water and freshly distilled methanol.

**Scheme 1.**
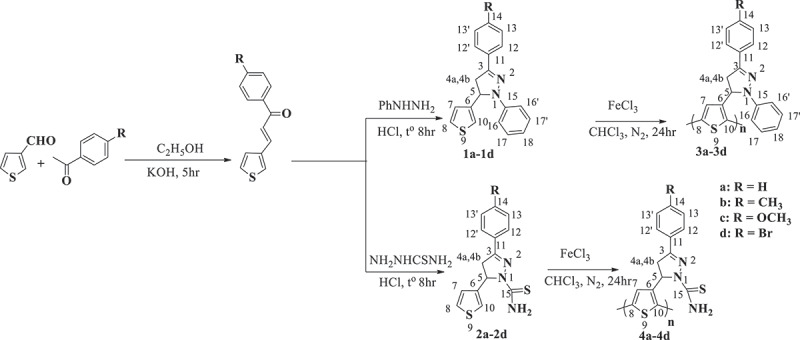
Synthesis of polythiophene containing polythiophene derivatives bearing pyrazoline.

The polymers were purified by Soxhlet extraction using methanol to remove oligomers and residual iron(III) chloride, and then with ethanol for 24 hours to remove unreacted monomers and oligomers. Finally, they were vacuum-dried for 1 day to yield dark red-coloured powders of the eight polythiophene derivatives containing pyrazoline **3a–3d, 4a–4d** with polymerization yields of 59–71%. The synthetic process of the new polythiophenes is described in Scheme 1.

### 2.4. Crystal structure determination (1b, 1d, 2b, 2d)

Crystals of **1b, 1d, 2b** and **2d** were obtained after several days from ethanol solutions and isolated by vacuum filtration. The crystals were washed with ethanol and dried under vaccuum. X-ray intensity data were collected at 293(2) K on an Agilent SuperNova diffractometer with Eos CCD detector using Mo-Kα radiation (λ = 0.71073 Å). The images were interpreted and integrated with CrysAlisPRO [37] in which the implemented absorption correction was applied. The structures were solved using Olex2 [38] with the ShelXT [39] structure solution program using Intrinsic Phasing and refined with the ShelXL [40] refinement package using full-matrix least-squares minimization on F^2^. Non-hydrogen atoms were refined anisotropically. Hydrogen atoms H8A and H8B of thioamide atom N8 in **2b** were located in a difference electron density map and subsequently refined freely. All other hydrogen atoms were placed at calculated and refined in the riding mode with isotropic temperature factors fixed at 1.2 times U_eq_ of the parent atoms (1.5 times U_eq_ for methyl groups). For **2b** and **2d**, the thiophene ring was refined in two orientations (occupancies 0.860(3):0.140(3) for **2b** and 0.763(5):0.264(5) for **2d**) using restraints on distances (DFIX), angles (DANG) and temperature factors (EADP), and for **2b** also on planarity (FLAT). The main crystallographic data together with refinement details are summarized in Table S1.

## 3. Results and discussion

### 3.1. Crystal structure determination of monomers 1b, 1d, 2b and 2d

**Figure 1.**
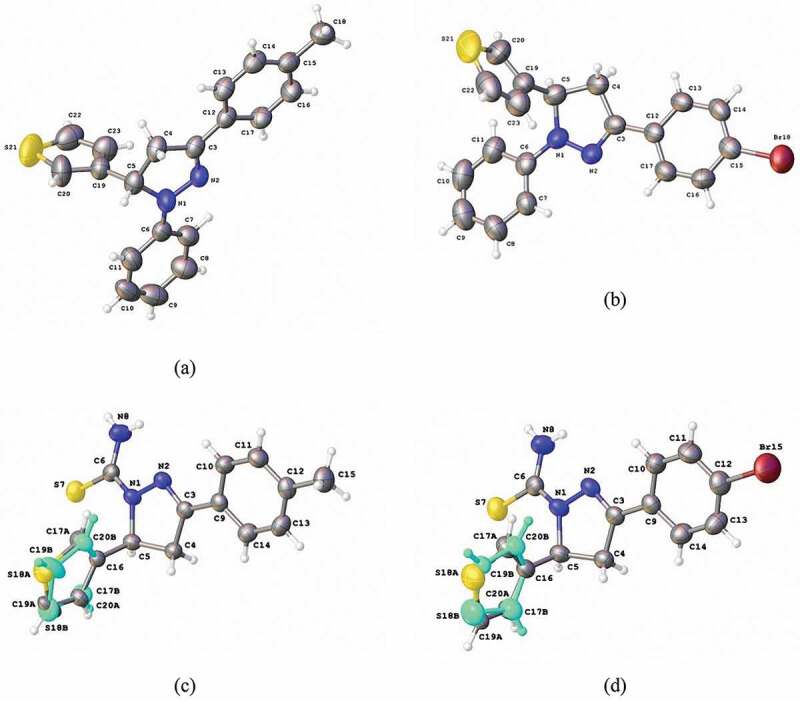
The molecular structure of monomers (a) 1b, (b) 1d, (c) 2b and (d) 2d with displacement ellipsoids drawn at the 50% probability level. For 2b and 2d the minor components of the disordered thiophene rings are shown in blue.

The X-ray single crystal study confirmed the structure of the synthesized monomers (Figure 1). Monomers **1b** and **1d** crystallize isomorphously in space group *P*2_1_/c. The overlay of both molecules gives an r.m.s. deviation of 0.0365 Å after inversion. The pyrazoline ring displays an envelope conformation with the thiophene bearing atom C5 as flap. The deviation of atom C5 from the best plane through the four other ring atoms is 0.243(4) and 0.218(1) Å for **1b** and **1d**, respectively. The thiophene and pyrazoline rings are oriented almost perpendicular to each other with a dihedral angle between the best planes through both rings of 81.28(14) and 82.9(2)° for **1b** and **1d**, respectively. Both phenyl rings do not deviate as much from the pyrazoline ring with dihedral angles between 6.92(11) and 16.88(19)°. The crystal packing of both compounds is characterized by the presence of C-H … π(phenyl) interactions and in the case of **1d** also a C-Br … π(thiophene) interaction [Br … *Cg*^i^ = 3.709(2) Å; *Cg* is the centroid of the thiophene ring; symmetry code: (i) -*x*, −1/2 + *y*, 3/2 – *z*].

Monomers **2b** and **2d** also crystallize isomorphously in space group *P*2_1_/c. The thiophene rings show rotational disorder with occupancies for the major component of 0.860(3) for **2b** and 0.763(5) for **2d**. The overlay of both molecules (major components) gives an r.m.s. deviation of 0.0374 Å. The conformation of the pyrazoline ring is twisted on the C4-C5 bond. The dihedral angle between the best planes through the major thiophene and pyrazoline rings is 81.2(2)° for **2b** and 81.7° for **2d**. The two other substituents again are rotated less out of the plane of the pyrazoline ring. For the thioamide substituent, the dihedral angle between both planes is 16.94(11)° for **2b** and 15.8(2)° for **2d**, for the phenyl substituent 12.27(11)° for **2b** and 13.1(2)° for **2d**. Next to the expected C-H … π(phenyl) and C-Br … π(thiophene) interactions, the crystal packing also shows N-H … S interactions.

### 3.2. Structure of polythiophenes derivatives containing pyrazoline

#### 3.2.1. FT-IR spectra of the polythiophene polymers

**Figure 2.**
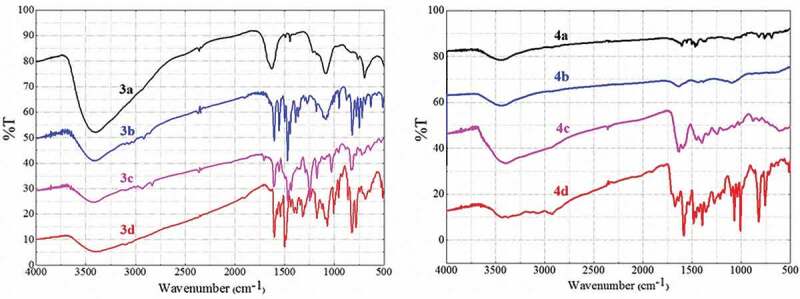
IR spectra of polymers 3a–3d (left) and polymers 4a–4d (right).

Based on FT-IR spectra, the chemical polymerization was confirmed since the synthesized polythiophenes had similar absorption bands as their precursor monomers. Moreover, the IR bands of the polythiophenes were obviously broadened compared with those of the monomers due to the wide chain dispersity of the obtained oligothiophenes and polythiophenes [41]. IR spectra of polymers still showed the strong bands at 1632–1604 cm^–1^ assigned to C = C bonds in the thiophene and the benzene rings (Figure 2, Table 1). For polymers **4a–4d**, there were strong and broad stretching bands belonging to the stretching vibration of N–H bonds at 3500–3400 cm^–1^ and bands belonging to the C = S bonds at 1213–1095 cm^–1^. For polymers **3b, 3c, 4b, 4c**, there was the additional appearance of bands at about 2939–2908 cm^–1^ attributed to saturated C–H bonds of – CH_3_ and – OCH_3_ groups.

**Table 1.** Some main vibrations in IR spectroscopy (cm^–1^) of the synthesized polymers

Polymer	υ_N–H_	υ_C–H aromatic_	υ_C=N, C=C_	υ_C–H out-of-plane_	υ_–CH3_,	υ_C=S_
3a	-	3058	1623	850	-	-
3b	-	3056	1605	823	2908	-
3c	-	3096	1606	829	2921	-
3d	-	3089	1604	825	-	-
4a	3397	-	1631	824	-	1157
4b	3409	3048	1609	875	2915,	1095
4c	3399	-	1585, 1631	880	2939,	1183
4d	3433	3075	1632	821	-	1213

**Figure 3.**
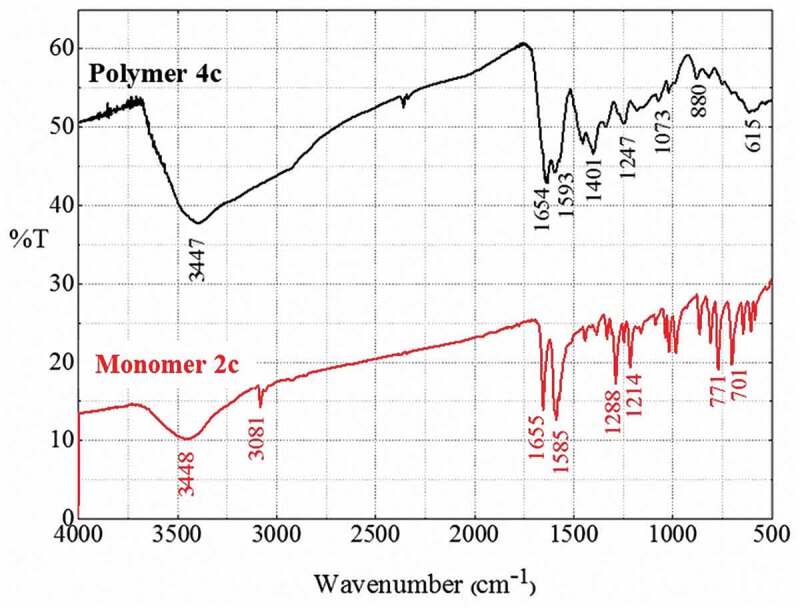
IR spectra of monomer 2c and polymer 4c.

Furthermore, decreasing intensities of the bands attributed to C–H bonds in the range of 3048 cm^–1^ to 3096 cm^–1^ were observed, indicating the disappearance of these bonds in the thiophene ring monomer during formation of the new C–C bonds of the polythiophene chain (Figure 3). A new absorption band with strong intensity at about 1630 cm^–1^ could be attributed to the conjugated C = C–C = C fragment in the polythiophene chain, which confirmed the successful polymerization and was further confirmed by Raman spectra. In addition, there was also a decrease in the intensity of the bands belonging to C–H out-of-plane vibration at 880–823 cm^–1^.

#### 3.2.2. ^1^H- NMR spectra of the polythiophene polymers

**Figure 4.**
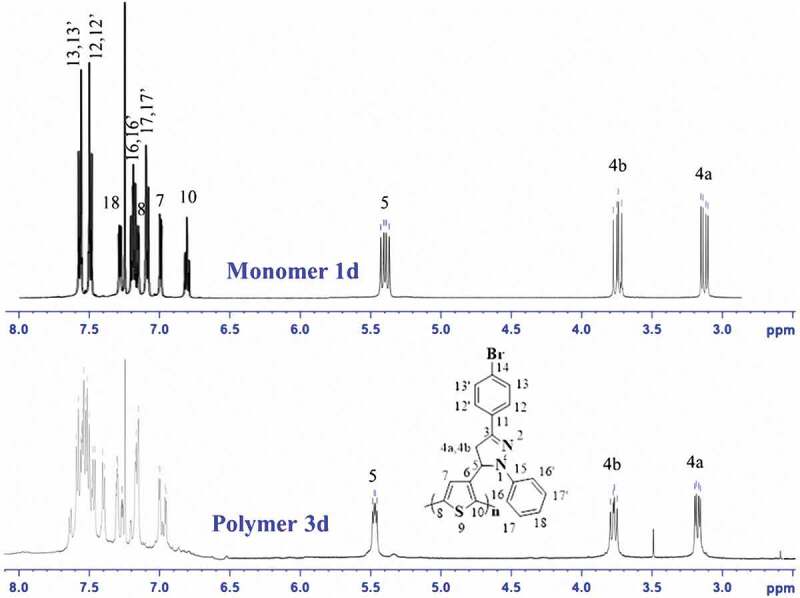
^1^H-NMR spectra of monomer 1d and polymer 3d.

The ^1^H-NMR spectrum (500 MHz, DMSO) of poly[3-(4-bromophenyl)-1-phenyl-5-thiophenyl-2-pyrazoline] **3d** is shown in Figure 4. The peaks were assigned by comparison with the spectra of monomer **1d** as follows: 5.3 ppm to the – CH proton in pyrazoline, 3.1 ppm and 3.7 ppm to the two methylene protons in pyrazoline, and all protons in the two benzene rings at about 6.91–7.09 ppm. The signals of the three protons at the 7-, 8- and 10- positions of the thiophene ring in monomer **1d** appeared at 6.98, 7.25 and 7.2 ppm, respectively. However, compared with the ^1^H-NMR spectra of the monomer, the peaks at 6.98 ppm and 7.2 ppm characterized as the resonances of protons at the 8- and 10-positions of the thiophene ring were almost absent in polymer **3d**. This was also a confirmation that the chemical oxidation polymerization was successful.

#### 3.2.3. UV-Vis spectra of the polythiophenes

**Figure 5.**
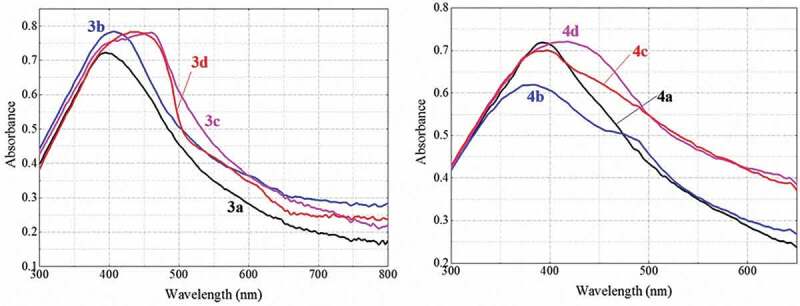
UV-Vis spectra of synthesized polymers 3a–3d (left) and polymers 4a–4d (right).

The UV-Vis spectra of the synthesized polythiophenes in the solid state are presented in Figure 5 and Table 2. Absorption spectra of the polymers all exhibit absorption bands in the typical wavelength region of 395**–**470 nm corresponding to the π→π* transition in the π–conjugated polythiophene [6,30,34]. Polymers **3c, 3d** and **4d** have longer absorption λ_max_ values at about 463, 431 and 415 nm, respectively, which show longer π–conjugated chains and better backbone coplanarity.

**Table 2.** Optical characteristics* of the synthesized polymers

Polymer	λ_max_ (nm)	λ_emission_ (nm)	I_emission_ (a.u)	Stokes shift (nm)
3a	395	550	9539	~155
3b	406	516	35,147	~110
3c	463	505	6918	~42
3d	431	527	46,410	~96
4a	392	536	31,093	~144
4b	415	546	24,691	~131
4c	395	549	20,760	~154
4d	385	646	39,687	~261

* λ_max_: Wavelength of the absorption maximum; λ_emission_: Wavelength of the emission maximum; I_emission_: Emission maximum intensity.

### 3.3. Morphology and properties of derivatives containing pyrazoline

Characteristics such as thermal stability, photoluminescence and conductivity of these polymers are related to properties of the main-chain thiophene rings and are discussed in this section.

#### 3.3.1. FE-SEM analysis of the polythiophenes

**Figure 6.**
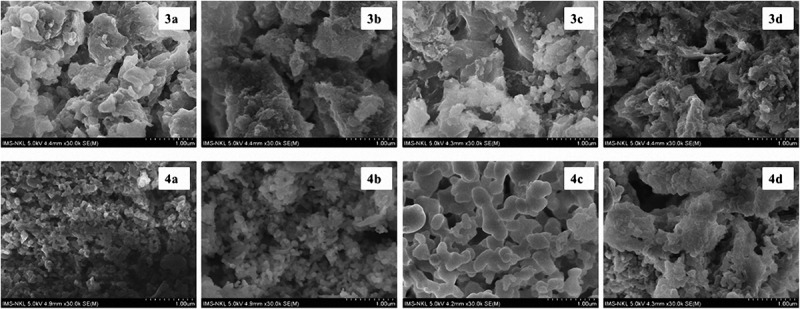
FE-SEM images of the synthesized polythiophenes.

Scanning electron microscope (SEM) images of the surface morphology of all synthesized polythiophenes are shown in Figure 6. These SEM images illustrate that for all polymers the formed particles are uniform and homogeneous with micro-sized dimensions. The morphological structure of polythiophenes **4a–4d** containing an – NH_2_CS group at the pyrazoline ring shows a uniform distribution and is porous, whereas particles of polythiophenes **3a–3d** containing only a phenyl group at the pyrazoline ring exhibit a higher order arrangement with higher adhesive states. This could be caused by longer π–conjugated polythiophene chains of the polymers **3a–3d**, as shown on TGA results.

#### 3.3.2. TGA analysis of the polythiophenes

**Figure 7.**
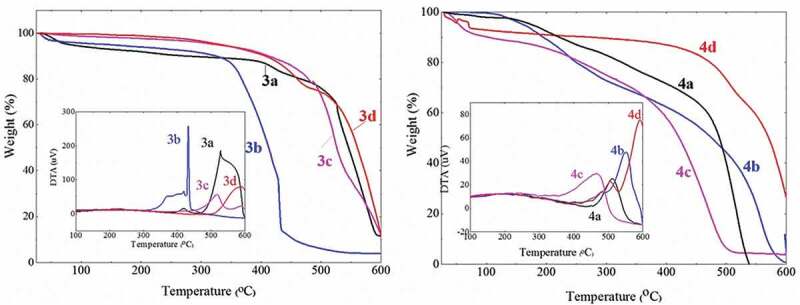
TGA and DTA (inset) thermograms of synthesized polymers 3a–3d (left) and polymers 4a–4d (right).

The side groups on the pyrazoline ring are expected to play an important role in improving the polythiophene thermal stability. Therefore, the thermal properties of the synthesized polythiophenes were studied through TGA/DTA thermal analyses (Figure 7 and Table 3). Firstly, all polymers have an average thermal stability in the air at about 431^○^C–591^○^C. Among them, polymers **3d** and **4d** have the best thermal stability with an endothermic temperature of 591^○^C and 590^○^C, respectively. This is consistent with the UV-Vis results which indicate that these two polymers have the longest π–conjugated polythiophene chains. Compared to our previous research, polythiophenes containing pyrazoline and benzene rings have a higher thermal stability than polythiophenes containing 1,2,4-triazoles, 2-thioxo-1,3-thiazolidin-4-one heterocycles or long hydrazone side groups [34,36,42]. This could be explained by the fact that the pyrazoline ring attached directly onto the conjugated polythiophene increases the coplanar arrangement in the polymer chain, leading to the effective π–conjugated polythiophene and thermal stability. Secondly, the thermal stability of polymers **3a–3d** is slightly better than that of polymers **4a–4d**. The reason could be that the benzyl group attached to the pyrazoline ring in polymers **3a–3d** significantly increases the polymer’s molecular weight, leading to an increase in thermal stability. However, the – NH_2_ groups capable of forming hydrogen bonds in polymers **4a–4d** increase their thermal stability.

**Table 3.** Thermal properties of the synthesized polymers

Polymer	Endothermic temperature	Remaining weight at 600°C (%)
3a	527	11.65
3b	431	4.09
3c	516	11.94
3d	590	9.8
4a	510	0
4b	551	0.67
4c	466	4.06
4d	504, 591	26.54

#### 3.3.3. Photoluminescence spectra of the polythiophenes

**Figure 8.**
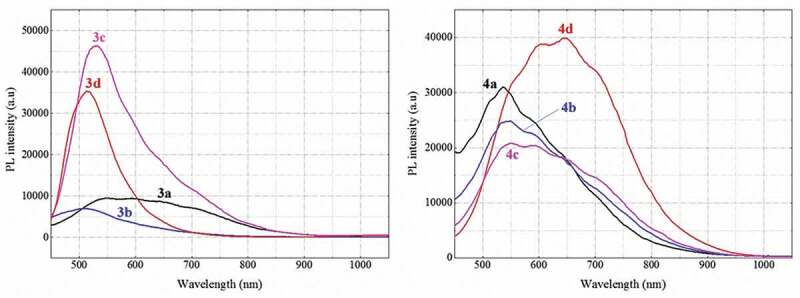
Photoluminescence spectra of synthesized polymers 3a–3d (left) and polymers 4a–4d (right).

The photoluminescence spectra of the polymers all have a maximum fluorescence emission in the range 505–550 nm. In particular, polymer **4d** has a maximum fluorescence emission at 646 nm (Figure 8 and Table 2). The two polymers **3d** and **4d** with a – Br benzene-substituted group have the strongest photoluminescence intensity of 46,410 a.u. and 39,687 a.u., respectively. However, the polymers with – OCH_3_ and – CH_3_ substituents have irregular fluorescence intensity. According to Radhakrishnan *et al*., the electron-repulsive and electron-attractive substituents affect the distance between LUMO and HOMO in polythiophene, which in turn affects the optical properties of polymer [30–32]. However, the distance between the substituents on the benzyl group of the polythiophene conjugated chain is quite long, so the electron-donating and electron-withdrawing substituents have negligible influence on the photoluminescence of the polymer.

Compared to some polythiophenes containing long hydrazone side groups [42,43], polythiophenes containing pyrazoline heterocycles have maximum fluorescence emission in the shorter wavelength region (about 50 nm) but have an emission intensity about 5–10 times higher. Therefore, depending on the application as electron transporting material, different polythiophene derivatives can be selected depending on their optical properties.

#### 3.3.4. Electrical conductivity of the polythiophenes

**Figure 9.**
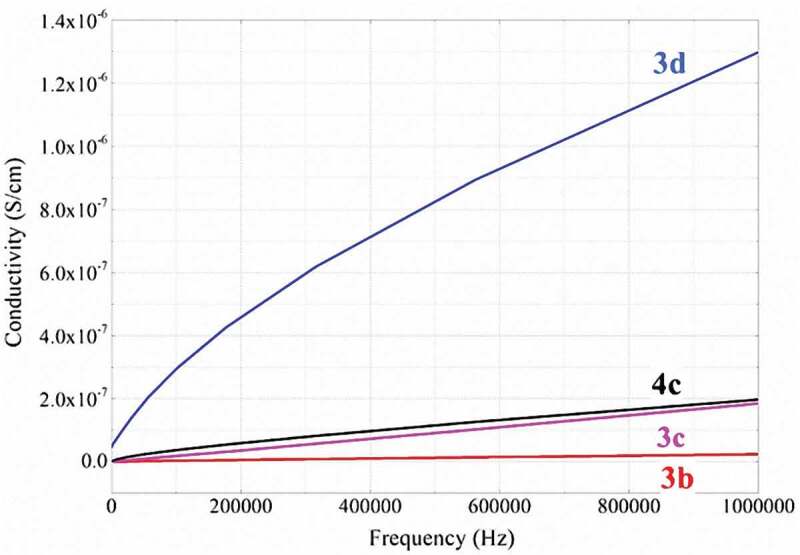
Conductivity of synthesized polymers 3b, 3c, 3d, 4c.

The electrical conductivity of the synthesized polythiophenes in the form of pressed pellets of 0.5 cm diameter was determined (Figure 9). The electrical conductivity is proportional to the increase in frequency from 0 Hz to 1 MHz at room temperature. At 1 MHz, polymer **3d** has a much better conductivity value (1.3 × 10^–6^ S/cm) than the other three polymers **3c, 3b, 4c**. However, it is difficult to explain this by the influence of the branch-chain substituents on the benzene and pyrazoline rings because these groups are too far away from the conjugated polythiophene. Based on the results of the morphological analysis (Figure 6), polymer **3d** has a tight arrangement with higher adhesion, so this polythiophene has a better conductivity than other polythiophenes.

In comparison with our previous studies, polythiophene **3d** containing a pyrazoline heterocycle has 10 to 100 times better electrical conductivity than polythiophenes containing 1,2,4-triazoles or various hydrazone side groups [34,42–47]. In addition, compared with typical undoped poly(3-hexylthiophene) having a moderately low conductivity of ~10^–8^ S/cm [51], the conductivity of polymer **3d** is about 100 times higher. However, the conductivity of the other polythiophenes **3c, 3b, 4c** is not too different from the previous studies.

## 4. Conclusions

A novel series of polythiophene derivatives containing a pyrazoline heterocycle were synthesized using chemical oxidation polymerization. The successful polymerization was verified by the comparison of the IR, ^1^H-NMR, Raman and UV-Vis spectral data of monomers and that of the resulting polymers. The spectroscopic analyses showed clear changes of some bands characterized for C = C–C = C conjugated polythiophene formation, as well as the disappearance of peaks assigned to two protons in the thiophene ring. The different substituents attached to the 1- and 3-positions on the pyrazoline side chain led to the differences in the optical properties, electrical conductivity, and thermal stability of the synthesized polythiophenes. Polythiophenes with the bromine substituents in the benzyl group **3d** and **4d** had the best electrical conductivity of about 1.3 × 10^–6^ S/cm, stable thermal stability until 590°C in the air and high fluorescence intensity (above 40,000 a.u.) at 505–550 nm. However, the different – C(NH_2_) = S and – C_6_H_5_ substituents attached at the 1-position of pyrazoline did not have much influence on the formation of the conjugated polymers, so the properties of the two polymer series **3a**–**3d** and **4a**–**4d** were not too different.

## Supplementary Material

Supplemental Material
