# Supplementary material for: Synthesis and characterization of some novel polythiophene derivatives containing pyrazoline
Source: Des Monomers Polym. 2022 Jun 9;25(1):136–47. doi: 10.1080/15685551.2022.2086413 (PMC9186369; doi:10.1080/15685551.2022.2086413)
Supplement: Supplemental Material [file TDMP_A_2086413_SM8523.docx]

**Synthesis and characterization of some novel polythiophene derivatives containing pyrazoline**

Vu Quoc Trung^1,*^, Tran Thi Thuy Duong^2^, Nguyen Thi Dua^1^, Nguyen Ngoc Linh^3^, Lai Dang Cuong^4^, Dao Phuong Thao^4^, Vo Khac Huy^5^, Nguyen Hoang Ha Phuong^6^, Nguyen Hien^1^, Duong Khanh Linh^1^, Vu Quoc Manh^3^, Nguyen Thuy Chinh^7,8^, Thai Hoang^7,8^, Luc Van Meervelt^9,*^

^1^Faculty of Chemistry, Hanoi National University of Education, 136 Xuan Thuy, Cau Giay, Hanoi 100000, Vietnam

^2^Tay Ho High School, 143 Phu Thuong, Tay Ho, Hanoi 100000, Vietnam

^3^Faculty of Pharmacy, Thanh Do University, Kim Chung, Hoai Duc, Hanoi 100000, Vietnam

^4^Bien Hoa Gifted High School, 86 Chu Van An, Phu Ly, Ha Nam Province, 400000, Vietnam

^5^HUS High School for Gifted Student, 182 Luong The Vinh, Thanh Xuan, Hanoi 100000, Vietnam

^6^Vinschool The Harmony High School, Nguyet Que 5, Viet Hung, Long Bien, Hanoi 100000, Vietnam

^7^Institute for Tropical Technology, Vietnam Academy of Science and Technology, 18 Hoang Quoc Viet, Cau Giay, Hanoi 100000, Vietnam

^8^Graduate University of Science and Technology, Vietnam Academy of Science and Technology, 18 Hoang Quoc Viet, Cau Giay, Hanoi 100000, Vietnam

^9^Department of Chemistry, KU Leuven, Biomolecular Architecture, Celestijnenlaan 200F, Leuven (Heverlee), B-3001, Belgium.

^*^Corresponding authors: [trungvq@hnue.edu.vn](mailto:trungvq@hnue.edu.vn), [luc.vanmeervelt@kuleuven.be](mailto:luc.vanmeervelt@kuleuven.be)

**Supplementary Information**

Table S1. Crystallographic data, details of data collection, and structure refinement parameters for **1b**, **1d**, **2b** and **2d**.

| Identification code | **1b** | **1d** | **2b** | **2d** |
| --- | --- | --- | --- | --- |
| Empirical formula | C_20_H_18_N_2_S | C_19_H_15_BrN_2_S | C_15_H_15_N_3_S_2_ | C_14_H_12_BrN_3_S_2_ |
| Formula weight | 318.42 | 383.30 | 301.42 | 366.30 |
| Temperature/K | 293(2) | 293(2) | 293(2) | 293(2) |
| Crystal system | monoclinic | monoclinic | monoclinic | monoclinic |
| Space group | *P*2_1_/c | *P*2_1_/c | *P*2_1_/c | *P*2_1_/c |
| a/Å | 5.9731(3) | 6.1662(2) | 14.2462(5) | 14.4370(6) |
| b/Å | 10.8513(5) | 10.7023(4) | 11.6955(4) | 11.7739(4) |
| c/Å | 26.2330(11) | 26.0760(9) | 9.0601(3) | 9.0409(4) |
| α/° | 90 | 90 | 90 | 90 |
| β/° | 95.693(4) | 96.866(3) | 102.757(4) | 101.935(4) |
| γ/° | 90 | 90 | 90 | 90 |
| Volume/Å^3^ | 1691.94(13) | 1708.48(10) | 1472.31(9) | 1503.55(11) |
| Z | 4 | 4 | 4 | 4 |
| ρ_calc_g/cm^3^ | 1.250 | 1.490 | 1.360 | 1.618 |
| μ/mm^‑1^ | 0.192 | 2.528 | 0.354 | 3.004 |
| F(000) | 672.0 | 776.0 | 632.0 | 736.0 |
| Crystal size/mm^3^ | 0.5 × 0.15 × 0.15 | 0.45 × 0.3 × 0.3 | 0.5 × 0.2 × 0.05 | 0.35 × 0.25 × 0.05 |
| Radiation | MoKα (λ = 0.71073 Å) | MoKα (λ = 0.71073 Å) | MoKα (λ = 0.71073 Å) | MoKα (λ = 0.71073 Å) |
| 2Θ range for data collection/° | 4.882 to 52.74 | 4.938 to 52.74 | 5.778 to 52.744 | 5.76 to 52.746 |
| Index ranges | -7 ≤ h ≤ 7, -13 ≤ k ≤ 13, -32 ≤ l ≤ 32 | -7 ≤ h ≤ 7, -13 ≤ k ≤ 13, -32 ≤ l ≤ 32 | -17 ≤ h ≤ 17, -14 ≤ k ≤ 14, -11 ≤ l ≤ 11 | -18 ≤ h ≤ 18, -14 ≤ k ≤ 14, -11 ≤ l ≤ 11 |
| Reflections collected | 33346 | 27178 | 23380 | 30012 |
| Independent reflections | 3472 [R_int_ = 0.0365, R_sigma_ = 0.0179] | 3498 [R_int_ = 0.0451, R_sigma_ = 0.0348] | 3012 [R_int_ = 0.0339, R_sigma_ = 0.0209] | 3072 [R_int_ = 0.0543, R_sigma_ = 0.0291] |
| Data/restraints/parameters | 3472/0/209 | 3498/0/208 | 3012/22/203 | 3072/20/194 |
| Goodness-of-fit on F^2^ | 1.044 | 1.027 | 1.081 | 1.041 |
| Final R indexes [I>=2σ (I)] | R_1_ = 0.0575, wR_2_ = 0.1623 | R_1_ = 0.0472, wR_2_ = 0.0950 | R_1_ = 0.0422, wR_2_ = 0.1041 | R_1_ = 0.0629, wR_2_ = 0.1422 |
| Final R indexes [all data] | R_1_ = 0.0721, wR_2_ = 0.1743 | R_1_ = 0.0818, wR_2_ = 0.1128 | R_1_ = 0.0520, wR_2_ = 0.1099 | R_1_ = 0.0909, wR_2_ = 0.1585 |
| Largest diff. peak/hole / e Å^-3^ | 0.20/-0.45 | 0.44/-0.47 | 0.44/-0.34 | 1.63/-1.32 |

Table S1. Crystallographic data, details of data collection, and structure refinement parameters for **1b**, **1d**, **2b** and **2d**.
